# Appearance of peanut agglutinin in the blood circulation after peanut ingestion promotes endothelial secretion of metastasis-promoting cytokines

**DOI:** 10.1093/carcin/bgab059

**Published:** 2021-07-05

**Authors:** Weikun Wang, Paulina Sindrewicz-Goral, Chen Chen, Carrie A Duckworth, David Mark Pritchard, Jonathan M Rhodes, Lu-Gang Yu

**Affiliations:** The Henry Wellcome Laboratory of Molecular and Cellular Gastroenterology, Institute of Systems, Molecular and Integrative Biology, University of Liverpool, Liverpool L69 3GE, UK

## Abstract

Peanut agglutinin (PNA) is a carbohydrate-binding protein in peanuts that accounts for ~0.15% peanut weight. PNA is highly resistant to cooking and digestion and is rapidly detectable in the blood after peanut consumption. Our previous studies have shown that circulating PNA mimics the actions of endogenous galactoside-binding protein galectin-3 by interaction with tumour cell-associated MUC1 and promotes circulating tumour cell metastatic spreading. The present study shows that circulating PNA interacts with micro- as well as macro-vascular endothelial cells and induces endothelial secretion of cytokines MCP-1 (CCL2) and IL-6 *in vitro* and *in vivo*. The increased secretion of these cytokines autocrinely/paracrinely enhances the expression of endothelial cell surface adhesion molecules including integrins, VCAM and selectin, leading to increased tumour cell-endothelial adhesion and endothelial tubule formation. Binding of PNA to endothelial surface MCAM (CD146), via N-linked glycans, and subsequent activation of PI3K-AKT-PREAS40 signalling is here shown responsible for PNA-induced secretion of MCP-1 and IL-6 by vascular endothelium. Thus, in addition to its influence on promoting tumour cell spreading by interaction with tumour cell-associated MUC1, circulating PNA might also influence metastasis by enhancing the secretion of metastasis-promoting MCP-1 and IL-6 from the vascular endothelium.

## Teaser

Peanut agglutinin is a carbohydrate-binding protein in peanuts that rapidly enters into the blood circulation after peanut consumption. This study shows that circulating peanut agglutinin interacts with the blood vascular endothelium and promotes endothelial secretion of metastasis-promoting cytokines. Frequent consumption of peanuts by cancer patients therefore might increase risk of metastasis.

## Background

Peanut agglutinin (PNA) accounts for about 0.15% of the weight of the common peanut *Arachis hypogaea* (1). PNA is a tightly globular protein and is highly resistant to cooking and digestion so its active form is extractable from the faeces of people who consume peanuts (2). Our previous studies have shown that PNA rapidly appears in the blood circulation after human consumption of peanuts (3). Up to 5 µg per ml of PNA were detectable 1 h after consumption of 200 g peanuts by healthy volunteers. PNA is a carbohydrate-binding protein (or lectin) and binds galactose-terminated glycans, in particular the oncofetal Thomsen-Friedenreich disaccharide (Galβ1-3GalNAcα-, the Thomsen-Friedenreich T or TF antigen) which is commonly overexpressed in >90% tumour cells (4) and is a natural ligand of the endogenous human galactoside-binding protein galectin-3 (5).

Galectin-3, expressed by many cell types especially epithelial and immune cells, is commonly over-expressed in cancer and promotes cancer progression and metastasis by interaction with galactoside-terminated cell surface glycans (6). Increased circulation of galectin-3 is frequently seen in cancer patients including colorectal, breast, lung, pancreatic cancer and melanoma (6,7). Patients with metastases often have higher circulating galectin-3 concentrations than those with localized tumours (7). Circulating galectin-3 actively promotes metastasis by direct interaction with circulating tumour cells (5,8) or the vascular endothelium (9,10). Binding of galectin-3 to TF on MUC1 of tumour cells induces MUC1 cell surface polarization and exposure of smaller cell surface adhesion molecules/ligands, leading to increase of tumour cell-endothelium adhesion (8) and tumour cell–cell homotypic aggregation (11), two important steps in metastasis. Binding of galectin-3 to CD146 (the melanoma cell adhesion molecule MCAM, also known as MUC18) on the surface of vascular endothelium promotes endothelial secretion of metastasis-promoting cytokines IL-6, G-CSF, GM-CSF and sICAM-1 (9). This effect was not seen following interaction between galectin-3 and tumour cells (9).

As the PNA glycan binding profile overlaps considerably with that of galectin-3, we speculated that circulating PNA might mimic the actions of circulating galectin-3, thus promoting cancer cell metastasis. In a recent report, we have shown that PNA, like galectin-3, also binds to TF expressed by MUC1 on tumour cells and induces MUC1 cell surface clustering, leading to increased adhesion of tumour cells to the vascular endothelium and also to increased tumour cell homotypic aggregation for formation of tumour micro-emboli (12). When tested *in vivo* in a mouse metastasis model, introduction of PNA into the circulation in mice enhances metastasis (12). This suggests that circulating PNA mimics the action of circulating galectin-3 by direct interaction with disseminating tumour cells in metastasis promotion.

In this study, we conducted a series of *in vitro* and *in vivo* studies to test whether circulating PNA can, like circulating galectin-3, also interact with the blood vascular endothelium and affect endothelial secretion of cytokines relevant to cancer metastasis.

## Materials and methods

### Materials

Asialo-fetuin (ASF), PNA, FITC-PNA, PNGase-F and non-enzymatic cell dissociation solution (NECDS) were from Sigma (Poole, UK). Calcein-AM cell labelling solution (C3099) was from Invitrogen (Paisly, UK). Human G-CSF, GM-CSF, IL-6, ICAM-1, MCP-1 and murine MCP-1 and IL-6 ELISA kits were from Peprotech (London, UK). Agarose- and FITC-conjugated PNA were from Vector Laboratories (Peterborough, UK). Human cytokine proteome profile array (ARY005), human phospho-kinase profile array (ARY003b), antibodies against CD146/MCAM (MAB932) and CD31/PECAM-1 (BBA7) were from R&D Systems (Abingdon, UK). Antibodies against AKT (9272), phosph-AKT (Thr308), PRAS40 (2610), phospho-PRAS40 (Thr246), STAT3 (79D7), phosph-STAT3 (Tyr705) were from New England BioLabs (Herts, UK). *O*-glycanase (Endo-glycosidases) was from Agilent (Santa Clara, CA).

### Cell lines

Human microvascular lung endothelial cells (HMVEC-Ls) and human macrovascular umbilical vein endothelial cells (HUVECs) were obtained from Lonza (Basel, Switzerland) and cultured in endothelial growth media EGM-2 and EGM with supplements (Lonza Bulletkits), respectively. Human colon cancer SW620 and HCT116 cells were obtained from the European Cell Culture Collections via the Public Health Laboratory Services (Porton Down, UK) and were cultured in Dulbecco’s modified Eagle’s medium (DMEM) (for SW620) and McCoy’s 5a medium (for HCT116), respectively. Human melanoma ACA19-cells was kindly provided by Dr. John Hilkens (Netherlands Cancer Institute) and cultured in DMEM medium. The cell lines were last authenticated by DNA profiling (Cell Line Authentication Facility, University of Liverpool) in 2020. All culture media contained 10% fetal calf serum, 100 U/ml penicillin, 100 µg/ml streptomycin and 2 mM glutamine.

### Human cytokine profile array

Confluent HMVEC-Ls cultured in 6-well plates were treated with 4 μg/ml PNA or 4 μg/ml control bovine serum albumin (BSA) for 24 h at 37°C. The culture media were collected and the concentrations of cytokines in the media were determined by the Cytokine Proteome Profiler Array in accordance to the manufacturer’s instruction.

### Human protein kinase profile array

Sub-confluent HUVECs were treated with PNA or BSA for 24 h. Cells were washed, lysed and analysed using the human Phosphor-kinase Proteome Profiler Array according to the instructions of the manufacturer. The array was visualized by Gel DocTM XR and analysed using Image Lab (Bio-Rad).

### Assessment of cytokine secretion from endothelial cells

Confluent monolayers of human micro-(HMVEC-Ls) or macro-(HUVECs) vascular endothelial cells were incubated with different concentrations of PNA or BSA, in the presence or absence of 20 µg/ml asialo-fetuin, 4 μg/ml galectin-3 or kinase inhibitors 5 µM Wortmannin (WM) or 20 µM LY294002 for different times. The culture media were collected and the concentrations of IL-6 and MCP-1 were determined by ELISA.

### Assessment of cytokine secretion in mice

Nine 7-week-old female Balb/c athymic mice, obtained from Charles River Laboratories (Margate, Kent, UK) and maintained and used in accordance with the animal care protocol approved by the UK Home Office regulations and University of Liverpool, were randomly divided into three equal groups and 10 µg PNA (5 µg/ml, assuming a 2 ml blood volume) was introduced by intravenous tail vein injection. Blood was obtained by cardiac puncture at 0, 24 and 48 h and the serum concentrations of MCP-1 and IL-6 were determined by ELISA.

### Determination of cell adhesion

Endothelial cells were cultured in two 96-well plates (A and B) for monolayer formation (1~2 days). Cells in plate A were treated with 4 µg/ml PNA or BSA (control) in the presence or absence of 20 µg/ml ASF, antibodies against IL-6 (5 ng/ml)/MCP-1 (40 ng/ml) for 24 h. The culture media were collected as conditioned medium.

Cancer cells were released from culture flasks by NECDS, washed with PBS, suspended to 5 × 10^6^/ml in serum-free medium and labelled with 10 μl/ml Calcein-AM for 30 min at 37°C. The cells were washed with PBS and re-suspended at 1 × 10^5^cells/ml with the conditioned medium obtained above. The culture medium in plate B was removed and replaced with the tumour cell suspension for 1 h at 37°C. The cell monolayer was gently washed twice with PBS before the adhesion tumour cells were measured by fluorescent microplate reader (TECAN infinite F200) at 485 nm excitation/535 nm emission.

### Analysis of endothelial cell surface adhesion molecules by flow cytometry

Sub-confluent endothelial cells were treated with 4 µg/ml PNA or BSA, in the absence or presence of antibodies against MCP-1 (20 ng/ml) and IL-6 (5 ng/ml) for 1 h at 37°C before they were released by NECDs. Cells were washed with PBS and fixed with 2% paraformaldehyde for 25 min. After washes with PBS, the cells were suspended to 10^6^ cells/ml in 5% goat serum/PBS for 30 min before incubation with antibodies (2.5 μg/ml) to CD44, integrinα5β1, integrinα5β3, E-selectin, VCAM or ICAM in 1% goat serum/PBS for 1 h. After 2 washes with PBS and application of FITC-conjugated secondary antibody, the cells were analysed by BD FACSAria III.

### Assessment of endothelial cell tubule formation

Ninety-six well plates were coated with 50 μl Matrigel matrix (BD Biosciences) at 37°C for 1 h. HUVECs were cultured in two plates (A and B) for monolayer formation (1~2 days). Cells in plate A were treated with 4 µg/ml PNA or BSA in the presence or absence of 20 µg/ml ASF, or neutralizing antibodies to IL-6 (5 ng/ml)/MCP-1 (40 ng/ml) for 24 h. The culture media were collected as conditioned medium. The cells in plate B were released by trypsin, washed with PBS and suspended at 10^5^ cells/ml with the conditioned medium from Plate A before application to the coated 96-well plates. After 24 h culture at 37°C, the cells were imaged and tubule length was calculated using Image J.

### SiRNA protein knockdown

Sub-confluent HUVECs cultured in 12-well plates were treated with 150 pmol MCAM, PECAM siRNA (siGenome Smart pool human MCAM and PECAM, Thermo Fisher) or control non-target siRNA (siGenome control siRNA #1, Thermo Fisher) in serum-free medium for 5 min before addition of 2.5 μl DharmaFect Transfection Reagent-4 (Thermo Fisher) in 100 µl serum-free medium for 20 min at room temperature. After addition of 800 μl EGM, cells were incubated for 16 h at 37°C before the medium was replaced with fresh medium for a further 24 h. The cells were either lysed by SDS-sample buffer for analysis of MCAM and PECAM expression by immunoblotting, or introduced with 4 μg/ml PNA or BSA for 24 h at 37°C before IL-6 and MCP-1 concentrations in the culture media were determined by ELISA.

### Immunoblotting

Sub-confluent HUVECs cultured in 12-well plates were treated with different concentrations of PNA for 1 h at 37°C. Cells were washed with ice-cold PBS, lysed in SDS-sample buffer and separated by electrophoresis. After transfer to nitrocellulose membrane, the blots were incubated with 1% BSA/PBS before they were probed with antibodies (1:1000) against pAKT, pPRAS40 or pSTAT3. After application of peroxidase-conjugated secondary antibody, the blots were developed using chemiluminescence and visualized with Gel DocTM XR. After imaging, the blots were stripped with stripping buffer (62.5 mM Tris-HCl, pH 6.7, 100 mM mercapto-ethanol, 2% SDS) and re-probed with antibodies against AKT, PRAS40 or STAT3.

### PNA-agarose affinity purification and mass spectrometry

HUVECs were lysed in lysis buffer (PBS, 0.5% Triton X-100, 0.5% NP-40 (v/v), protease inhibitors) and sonicated 3 × 20 s on ice. After centrifugation at 16 000*g* for 10 min at 4°C, the cleared lysate was applied to PNA-agarose. After three washes with PBS, the bound proteins were eluted with 0.2 M lactose/PBS. The eluate was dialysed at 4°C for 24 h against H_2_O. The purified samples were freeze-dried and analysed by SDS-PAGE followed by silver staining.

A proportion of the freeze-dried purified sample was also analysed by LC/MS-MS (QExactive quadrupole-Orbitrap mass spectrometer coupled to a Dionex Ultimate 300 RSLC nano-liquid chromatograph) with the same protocol as we previously described (10). Raw data files were searched in Proteome Discoverer (v1.4) against the UniProt human reviewed database (20 187 sequences) using the Mascot search engine. A precursor mass tolerance of 10 ppm and a fragment ion mass tolerance of 0.01 Da were applied. A peptide false discovery rate of 1–5% was applied.

### Immunofluorescence and confocal microscopy

Sub-confluent HUVECs cultured on glass coverslips in 12-well plates were fixed with 4% paraformaldehyde. After incubation with 2% BSA/PBS for 1 h, the cells were incubated with 4 μg/ml FITC-PNA, antibodies to MCAM or PECAM (1:1000) in 1% BSA/PBS for 1 h at room temperature. After three washes with PBS, the cells were incubated with Alexa fluor 643-conjugated secondary antibody for 1 h at room temperature. The cells were washed five times with PBS before being mounted with DAPI-containing fluorescent mounting media (Vector Laboratories, Burlingame, CA) and analysed by confocal microscopy (Marianas SDC, 3i Imaging).

### Protein de-glycosylation

Sub-confluent HUVECs were lysed with lysis buffer (1% Triton X-100 in PBS) on ice. After centrifugation at 4500 rpm for 15 min, the supernatant was collected and three 1 ml supernatant aliquots were incubated each with 100 µl PNA-agarose beads (pre-washed with PBS) at 4°C overnight on a roller. The beads were washed twice with PBS before incubation with 5U PNGase-F, 10mM *O*-glycanase, or PBS at 37°C overnight. After three washes with PBS, PNA-bound proteins were released by SDS-sample buffer and analysed by immunoblot

### Statistical analysis

One-way analysis of variance (ANOVA) followed by Bonferroni correction (SPSS 24t) was used for multiple comparisons between groups. Differences were considered significant when *p* < 0.05.

## Results

### PNA induces secretion of MCP-1 and IL-6 from vascular endothelial cells *in vitro* and *in vivo*

**Figure 1.**
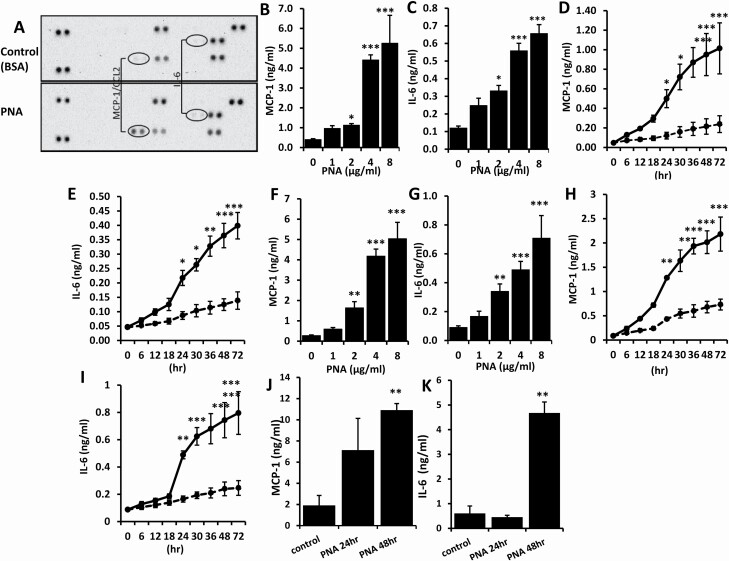
PNA induces MCP-1 and IL-6 secretion from micro- and macro-vascular endothelial cells *in vitro* and in mice. **(A**) HMVEC-Ls were treated with 4 μg/ml PNA or BSA for 24 h. The levels of cytokines in the culture media were analysed by cytokine profile array. PNA induces dose- (**B**, **C** and **F**, **G**) and time- (**D**, **E** and **H**, **I**) dependent increase of MCP-1 (**B**, **D** and **F**, **H**) and IL-6 (**C**, **E** and **G**, **I**) secretion from micro-vascular HMVEC-Ls (**A**–**E**) and macro-vascular HUVECs (**F**–**I**). (**J** and **K**) Nine mice were injected intravenously with 10 μg PNA and the blood from three mice at 0, 24 and 48 h were obtained and serum concentrations of MCP-1 and IL-6 in the blood were analysed. Data are presented as Mean ± SD of three independent experiments, each in triplicate. *p < 0.05, **p < 0.01, ***p < 0.001.

To determine the influence of PNA on cytokine secretion from vascular endothelial cells, human lung micro-vascular endothelial cells (HMVEC-Ls) were treated with 4 μg/ml PNA, a concentration seen in the venous blood of healthy volunteers who ingested 200 g peanuts (3), and the concentrations of 36 common cytokines were analysed using human cytokine profile arrays. In comparison to the cells treated with control BSA, treatment of the cells with PNA for 24 h resulted in 12- and 4-fold increases of MCP-1 and IL-6 secretion by HMVE-Ls (Figure 1A). This effect was PNA dose- (Figure 1B and C) and time- (Figure 1D and E) dependent. Similar PNA treatment was applied to macrovascular endothelial cells—human umbilical vein endothelial cells (HUVECs). Similar dose- (Figure 1F and G) and time- (Figure 1H and I) dependent increases of MCP-1 and IL-6 secretion were observed following PNA treatment. A 13- and 3-fold increase of MCP-1 and IL-6 secretion, respectively, was seen after 24 h treatment with 8 μg/ml PNA.

To assess its effects *in vivo*, PNA (10 µg) was intravenously injected into mice and the concentrations of MCP-1 and IL-6 in the blood serum were measured. PNA increased secretion of MCP-1 (Figure 1J) and IL-6 (Figure 1K). Approximately 6- and 7-fold increases of MCP-1 and IL-6 were detected after 48 h PNA inoculation.

### PNA-induced cytokine secretion enhances expression of endothelial cell surface adhesion molecules which promote cancer cell-endothelial cell adhesion.

**Figure 2.**
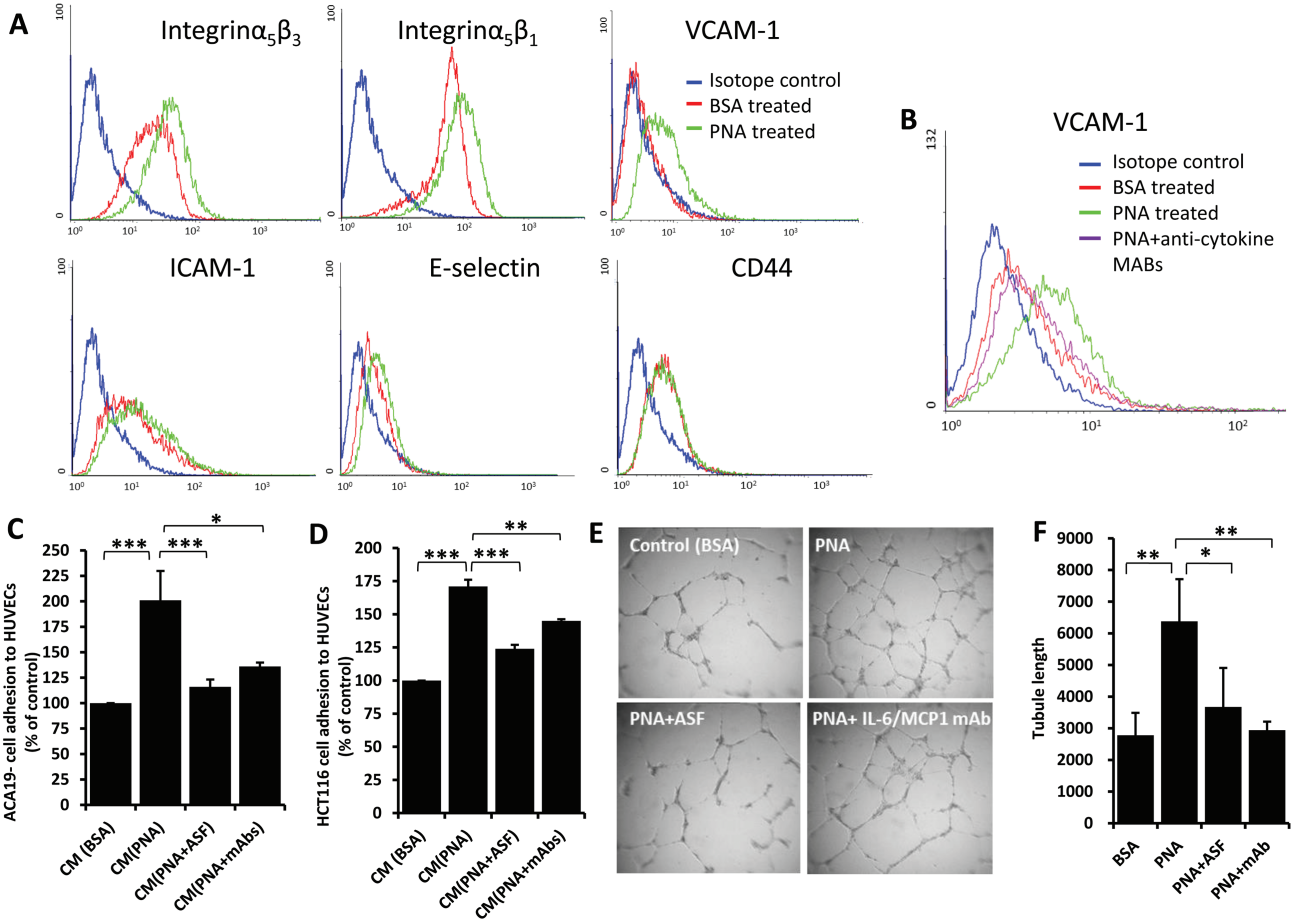
PNA-induced cytokine secretion increases cancer cell-endothelial adhesion and endothelial tubule formation. (**A** and **B**) PNA-induced cytokine secretion increases the expression of endothelial cell surface adhesion molecules. HUVECs were treated with 4 μg/ml PNA or BSA without (**A**) or with antibodies against MCP-1 and IL-6 (**B**) for 24 h. The expressions of cell surface integrinα5β3, integrinα5β1, VCAM-1, ICAM-1, E-selectin or CD44 were analysed by flow cytometry. (**C** and **D**) PNA-induced cytokine secretion increases cancer cell-endothelial adhesion. Conditional media (CM) from HUVECs treated with PNA or BSA in the presence or absence of ASF or neutralizing antibodies to IL-6 and MCP-1 for 24 h were used to assess adhesion of ACA19- (**C**) and HCT116 (**D**) cells to fresh HUVECs. (**E** and **F)** PNA-induced cytokine secretion promotes angiogenesis. HUVECs cultured on matrix gel were treated with 4 μg/ml PNA or BSA in the presence or absence of 20 µg/ml ASF or neutralizing antibodies to IL-6 (5 ng/ml) and MCP-1 (40 ng/ml) for 24 h before endothelial tubular length was quantified (**E** and **F**). Representative images are shown in (**E**). Data are presented as Mean ± SD of three independent experiments, each in triplicate. *p < 0.05, **p < 0.01, ***p < 0.001.

It was found that treatment of HUVECs with PNA (4 µg/ml) for 24 h increased the expression of cell surface Integrinα5β1 (by 29%), Integrinα5β3 (33%) E-selectin (20%), VCAM-1 (45%) and ICAM-1 (13.3%), but not CD44 (Figure 2A). The presence of neutralizing antibodies against IL-6 and MCP-1 reduced the PNA-mediated increase of VCAM-1 expression by 79% (Figure 2B), indicating the increased appearance of these cell surface adhesion molecules by PNA are consequence of MCP-1 and IL-6 secretion.

To test whether the increased appearance of these cell surface adhesion molecules by PNA-mediated MCP-1 and IL-6 secretion affects tumour cell-endothelial interaction, HUVECs were treated with PNA, without or with PNA-binding glycoprotein ASF or antibodies to MCP-1 and IL-6. The culture medium (conditioned medium) was collected and used to assess adhesion of human cancer cells to fresh HUVECs. Because PNA can enhance tumour cell-endothelial cell adhesion directly by interaction with cell surface MUC1 on tumour cells (12), MUC1-negative human colon cancer HCT116 and melanoma ACA19-cells were used here to avoid the PNA-MUC1-mediated influence on cancer cell interaction. It was found that in comparison to the medium obtained from BSA-treated cells, conditioned medium from PNA-treated HUVECs caused significant increase of adhesion of both ACA19- and HCT116 cells (Figure 2C and D). Such an increase of cancer cell adhesion by PNA did not occur in the presence of PNA-binding inhibitor ASF. A similar effect was also observed in HMVEC-Ls (data not shown). These results indicate that PNA-mediated increases of MCP-1 and IL-6 secretion enhance the expression of endothelial cell surface adhesion molecules that increase cancer-endothelial cell adhesion. The reduction of tumour cell adhesion in the presence of neutralizing antibodies to MCP-1 and IL-6 supports the involvement of these molecules, either on endothelial or tumour cells, in tumour cell adhesion.

### PNA-induced cytokine secretion promotes endothelial tubule formation

Treatment of HUVECs with PNA was seen to significantly increase HUVEC tubule formation (Figure 2E and F). The presence of ASF or neutralizing antibodies to IL-6 and MCP-1 largely abolished this effect. This indicates that the PNA-mediated increase of MCP-1 and IL-6 secretion promotes endothelial tubule formation, a component in the multi-stepped process of angiogenesis.

### PNA and galectin-3 partly compete with each other for endothelial induction of cytokine secretion

**Figure 3.**
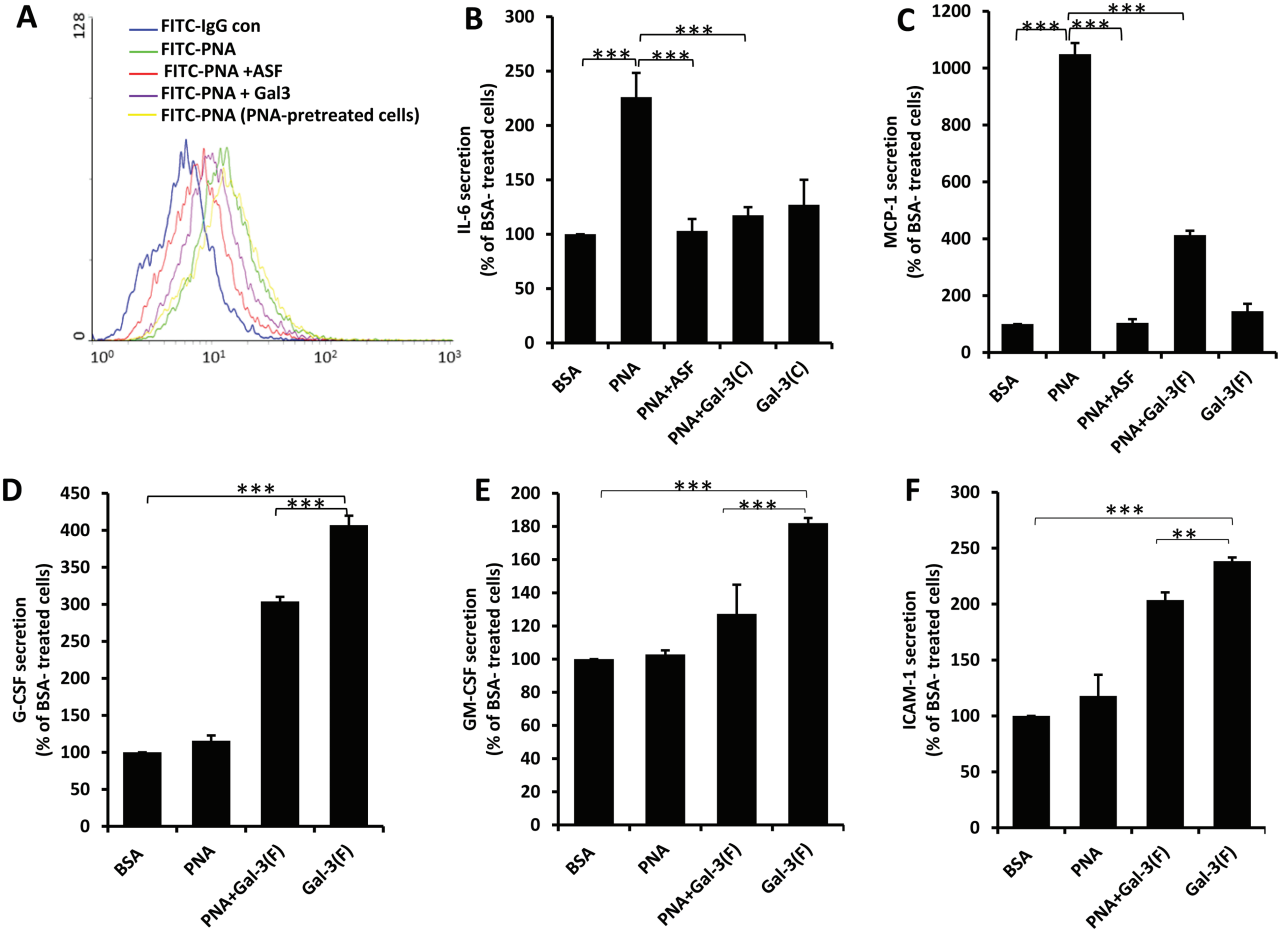
Partial competition between PNA and galectin-3 on cytokine secretion in endothelial cells. (**A**) HUVECs were treated with 4 µg/ml BSA or PNA for 1 h, fixed and applied with FITC-PNA, without or with 20 µg/ml ASF, 4 µg/ml galectin-3 before analysis by flow cytometry. (**B**–**F**): HUVECs were treated with 4 μg/ml PNA or BSA in the presence or absence of 20 μg/ml ASF, 4 μg/ml full-length galectin-3 (Gal-3F), or C-terminal galectin-3 (Gal-3C) for 24 h before the concentrations of the cytokines in the culture medium were analysed. Data are presented as Mean ± SD of three independent experiments, each in triplicate. *p < 0.05, **p < 0.01, ***p < 0.001.

PNA and galectin-3 both recognize galactoside-terminated glycans. Our earlier study has shown that galectin-3 enhances endothelial secretion of four cytokines including IL-6 (9). As secretion of IL-6 by endothelial cells was also seen to be increased by the presence of PNA in this study, we analysed the relationship of cytokine secretion mediated by PNA and galectin-3. The presence of PNA inhibitor ASF 20 µg/ml caused ~60% reduction of PNA (4 µg/ml) binding to the HUVEC surface, whereas the presence of galectin-3 (4 µg/ml) resulted in 25% reduction of PNA binding (Figure 3A). This suggests that PNA and galectin-3 share some but not all cell surface receptors. In support of this, PNA-mediated increase of IL-6 secretion was abolished by the presence of N-terminally truncated galectin-3 (Gal3C, which binds to galectin-3 carbohydrate ligands but cannot induce receptor clustering) (13) as well as by ASF (Figure 3B) but PNA-mediated secretion of MCP-1, whose secretion was not affected by galectin-3 (9), was only partially inhibited, but not abolished, by the presence of even the full-length form of galectin-3 (Gal3F) (Figure 3C). Moreover, the galectin-3-mediated secretion of G-CSF, GM-CSF and ICAM-1 was also only partly reduced by the presence of PNA (Figure 3D–F).

### PNA binding to cell surface MCAM (CD146), via N-linked glycan on MCAM, is responsible for PNA-mediated endothelial cytokine secretion

**Figure 4.**
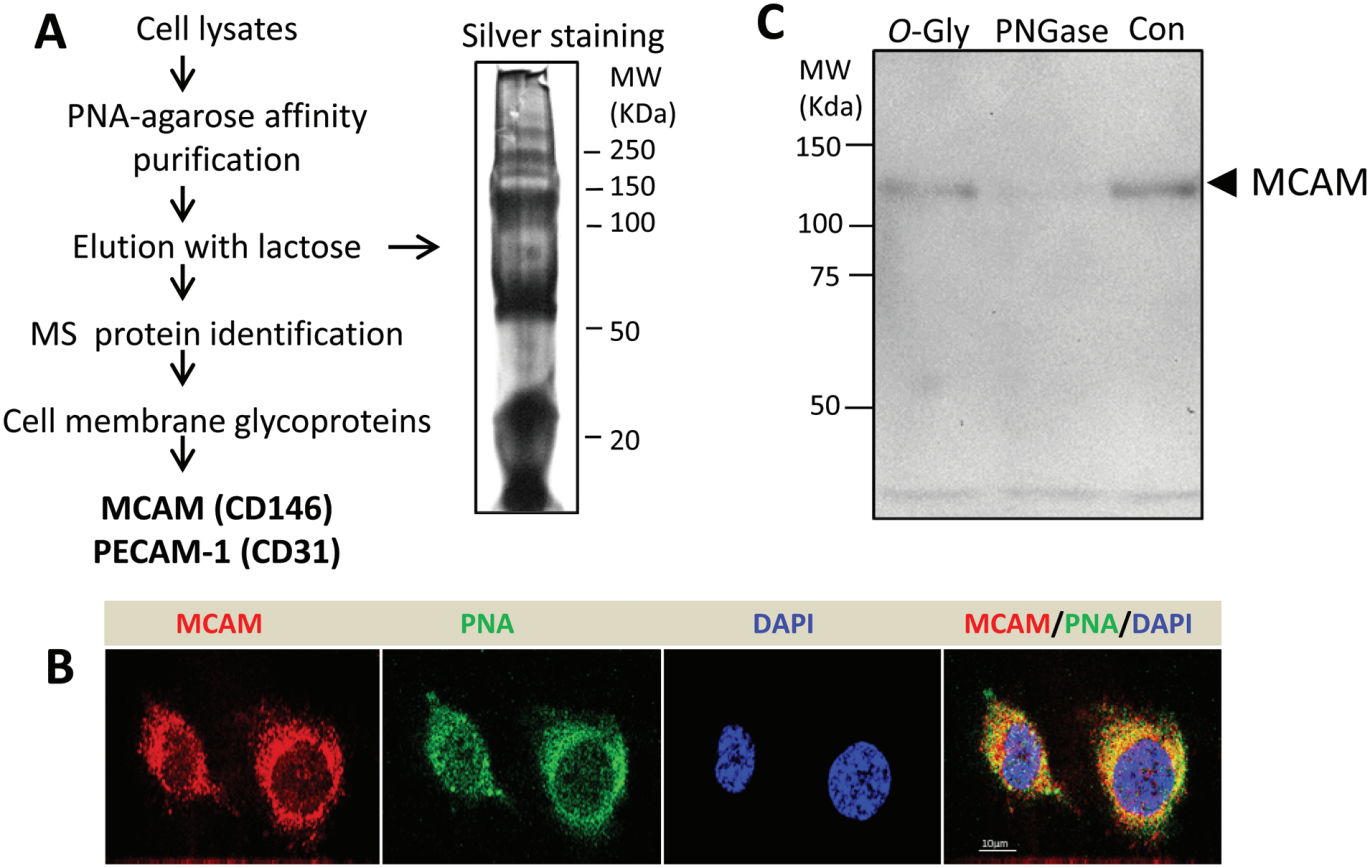
Identification of PNA binding ligands on endothelial cell surface. (**A**) PNA-agarose affinity purification followed by mass spectrometry analysis reveals MCAM and PECAM as two major cell surface glycan to be extracted by PNA affinity purification from HUVECs (insert shows silver staining of the eluted proteins eluted from PNA-agarose and separated by SDS-PAGE). (**B**) Representative confocal microscopy images show co-localization of PNA with MCAM on HUVEC cell surface. (**C**) MCAM immunoblots of PNA-agarose precipitates, without or with pre-treatment of the proteins with *N*- (PNGase-F) or *O*-glycanase. Treatment of the PNA precipitates with *N*-, but not *O*-glycanase, substantially reduces MCAM in the precipitates.

PNA-agarose affinity purification followed by mass spectrometry identified MCAM (CD146) and PECAM (CD31) as the major cell membrane glycoproteins to be extracted by PNA in HUVECs (Figure 4A). Analysis by confocal microscopy showed co-localization of PNA with MCAM (Figure 4B). The expression of PECAM in HUVECs was very weak and no co-localization of PNA with PECAM was observed (not shown). Pre-treatment of HUVECs with *N*-glycanase, but not *O*-glycanase, markedly reduced PNA-agarose-extraction/precipitation of MCAM (Figure 4C), whereas no PECAM was detected in the PNA-agarose precipitates nor was it influenced by *N*- or *O*-glycanase treatment (not shown).

**Figure 5.**
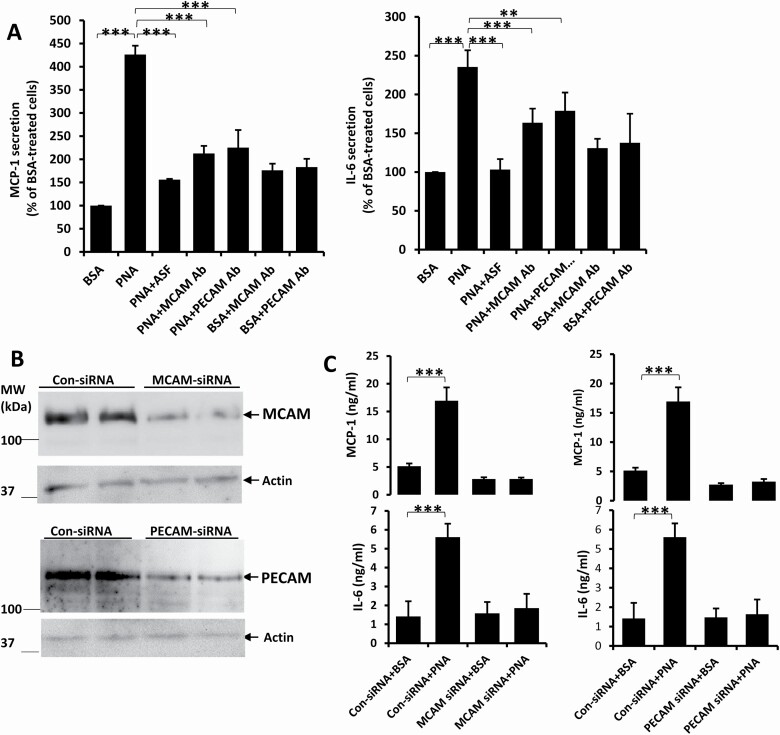
MCAM and PECAM are both involved in PNA-mediated cytokine secretion. The presence of ASF or neutralizing antibodies to MCAM or PECAM reduced PNA-mediated secretion of MCP-1 and IL-6 (**A**) from HUVECs. siRNA suppression of MCAM and PECAM expression (**B**) each abolished PNA-induced secretion of MCP-1 and IL-6 (**C**). Data are presented as Mean ± SD of three independent experiments, each in triplicate. **p < 0.01, ***p < 0.001.

Presence of neutralizing antibodies to either MCAM or PECAM was seen to partly, but not completely, suppress PNA-mediated secretion of MCP-1 and IL-6 (Figure 5A) from HUVECs. siRNA suppression of MCAM or PECAM expression (Figure 5B) each however almost abolished PNA-mediated MCP-1 and IL-6 secretion (Figure 5C). These suggest that MCAM and PECAM are both involved in PNA-mediated cytokine secretion.

### PNA-mediated cytokine secretion involves activation of PI3K-AKT-PRAS40 signalling

**Figure 6.**
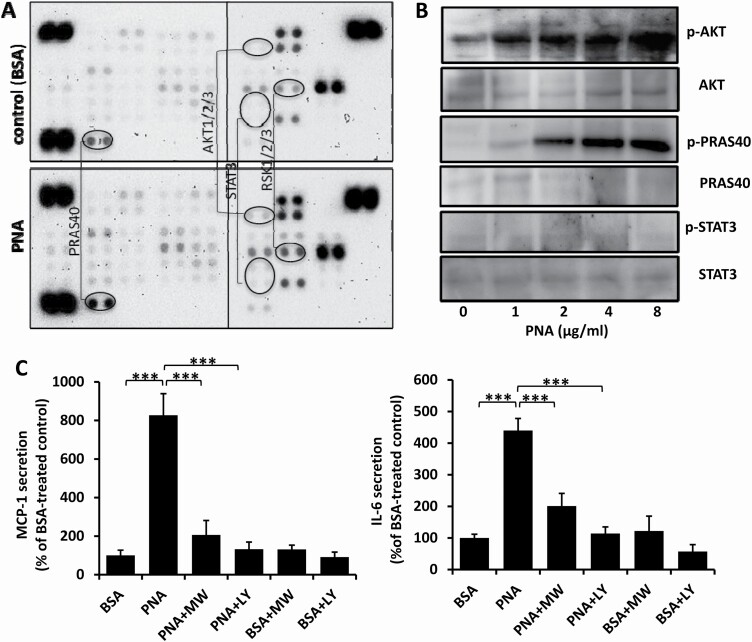
PNA-mediated cytokine secretion involves activation of PI3K-AKT-PRAS40 signalling. Profile array analysis shows that treatment of HUVECs PNA increased phosphorylation of AKT, STAT3, RSK and PRAS40 (*A*). PNA induces dose-dependent increase of AKT and PRAS40 phosphorylation but not STAT3 (**B**). The presence of PI3K inhibitors Wortmannin (WM) and LY294002 (LY) inhibit PNA-mediated secretion of MCP-1 and IL-6 (**C**) from HUVECs. Data are presented as Mean ± SD of three independent experiments. ***p < 0.001.

HUVECs were treated with PNA and the phosphorylation status of cell signalling proteins analysed by protein kinase profile array. Among the 43 signalling proteins in the array, four proteins AKT, STAT3, RSK and PRAS40 showed increased phosphorylation (Figure 6A). To determine the involvement of these cell signalling proteins in PNA-mediated cytokine secretion, the cells were treated with different concentrations of PNA and the phosphorylation status of these proteins was analysed by immunoblotting. PNA treatment resulted in dose-dependent increases of AKT- and PRAS40-phosphorylation but not STAT3 (Figure 6B). The presence of Wortmannin and LY294002, which are both inhibitors of one of the key AKT activators PI3K, significantly inhibited PNA-mediated cytokine secretion, particularly MCP-1 (Figure 6C). It is known that AKT activation is one of the key downstream signalling events involved in cell surface MCAM and PECAM activation (14,15), whereas PRAS40 is an AKT substrate (16). Together, these results suggest that activation of PI3K-AKT-PRAS40 signalling is critical in signalling transduction of PNA-mediated cytokine secretion.

## Discussion

In this study PNA, at concentrations found in the blood shortly after consumption of peanuts, interacts with blood vascular endothelial cells and induces secretion of MCP-1 and IL-6 *in vitro* and *in vivo*. This effect is mediated via PNA binding to *N*-linked glycans on MCAM on the endothelial cell surface and subsequent activation of PI3-AKT-PRAS40 signalling. PNA-mediated release of MCP-1 and IL-6 causes autocrine/paracrine interactions with the endothelium and enhances the expression of a number of cell surface adhesion molecules which promote endothelial-tumour cell adhesion and endothelial tubule formation.

MCP-1 (also known as chemokine C–C motif ligand 2, CCL2) is a CC chemokine family member. It is secreted by several cell types including endothelial cells, fibroblasts, monocytes, and cancer cells. MCP-1 governs the recruitment of monocytes, macrophages and other inflammatory cells, via binding to its receptor CCR2, in inflammation. Although MCP-1 was first described as a cytokine that regulates inflammation, subsequent studies have revealed its role in the promotion of cancer metastasis (17,18). Binding of MCP-1 to its receptor CCR2 on tumour cells enhances tumour cell proliferation, migration, invasion, and angiogenesis (19,20), partly through activation of the ERK signalling cascade and secretion of proteases (21). Elevated circulating concentrations of MCP-1 correlate with tumour grade, disease progression and metastasis in patients with breast, colorectal, prostate, melanoma, gastric and ovarian cancers (22). Administration of an antibody against MCP-1 has shown promising results in metastasis reduction in early phase clinical trials (18,23).

IL-6 is a multifunctional cytokine secreted by immune, endothelial and epithelial cells. It plays diverse roles in inflammation and immune reactions through binding to its cell surface receptor IL-6R. Rapid production of IL-6 contributes to host defense during infection and tissue injury, but excessive synthesis of IL-6 and dysregulation of IL-6R signalling is often involved in disease pathology such as tumour progression and metastasis (24). Binding of IL-6 to its receptor IL-6R in cancer cells activates multiple cellular signalling pathways including JAK/STAT, PI3K/Akt (25), leading to enhanced expression of a variety of promoters of tumour growth, angiogenesis and cancer progression. For example, IL-6–IL-6R interaction stimulates the release of VEGF and bFGF that promote angiogenesis (26). IL-6 activates Stat-3 signalling in regulator T cells and helps tumour cells escape immune surveillance (27). IL-6-mediated activation of Stat-3 signalling in inflammatory cells also leads to transcriptional activation of NF-kB signalling with consequential promotion of additional IL-6 and IL-8 secretion, thus generating a positive feedback loop between immune cells and tumour cells that further stimulates tumour progression (28). IL-6 also aids recruitment of circulating tumour cells back into their primary tumour site and accelerate tumor growth and angiogenesis (29). High levels of circulating IL-6 in the blood correlate with metastasis and poor prognosis in several types of cancers (30). Clinical trials using anti-IL-6 antibodies or IL-6 inhibitors, alone or in combination with other therapies, have demonstrated benefit in patients’ survival (31,32). Given the various roles of IL-6 and MCP-1 in promotion of cancer progression and metastasis, an increased secretion of IL-6 and MCP-1 by the vascular endothelium in response to the presence of PNA likely will have a broad and positive impact on metastasis. This is clearly supported by the demonstration of increased cancer-endothelial adhesion and angiogenesis (Figure 2) by PNA-mediated secretion of these cytokines.

MCAM (CD146) and PECAM-1 (CD31) were found to be the major endothelial cell surface glycoproteins to be extracted by PNA-agarose affinity purification in this study. MCAM and PECAM-1 are both members of immunoglobulin superfamily of cell adhesion molecules. They are both heavily glycosylated, with MCAM containing eight and PECAM containing nine potential N-glycosylation sites, and carbohydrates constitute ~35% and ~40% of the molecular weights of MCAM and PECAM, respectively (33,34). Both molecules are involved in endothelial cell homotypic (endothelial–endothelial) and heterotypic cell interactions (e.g. endothelial–leukocytes) and participate in endothelial cell activity and angiogenesis (35). After ligand engagement, MCAM and PECAM can both mediate outside-in signalling via their intracellular domains (36,37). For example, in response to binding by VEGFR-2, MCAM activates p38 and AKT signalling and increases endothelial cell migration (38). Binding of MCAM by galectin-3 induces MCAM cell surface dimerization and subsequent activation of AKT signalling that leads to secretion of IL-6, G-CSF, GM-CSF and ICAM-1 by vascular endothelial cells (10). Binding of MCAM by Netri-1 has been reported to activate ERK signalling and enhances endothelial cell proliferation, adhesion, migration and angiogenesis. (39). PECAM activation, often through PECAM–PECAM or PECAM–cell surface molecule ligation, regulates a number of biological processes such as angiogenesis and cell migration (40). Like MCAM, PECAM can also interact with VEGFR in endothelial cells, leading to activation of AKT signalling and secretion of cytokines such as IL-6 (41).

In this study, knockdown of MCAM or PECAM expression each substantially prevented PNA-mediated secretion of MCP-1 and IL-6 (Figure 5). This indicates that both MCAM and PECAM are involved in PNA-mediated actions. The discovery that introduction of PNA led to strong cell surface co-localization of PNA with MCAM, but not PECAM and that only MCAM, but not PECAM, was identified in PNA co-precipitation (Figure 4) indicate that MCAM is a direct PNA-binding ligand. It is possible that PECAM may form a functional complex with MCAM in signalling transduction in PNA-mediated cytokine secretion. The observation that reduction of MCAM *N*-glycosylation decreases PNA binding to MCAM further confirms a direct interaction of PNA with MCAM (via binding of PNA to the N-linked glycans of MCAM).

It was found that PNA-induced cytokine secretion could be substantially, but not completely, inhibited by the presence of galectin-3 (Figure 3). This suggests that PNA and galectin-3 share substantial, but not all, binding ligands on endothelial cell surface. The observation that presence of galectin-3 caused ~43% reduction of PNA binding to the endothelial cells (Figure 3A) supports this. This is in keeping with the discovery that MCAM is critical in galectin-3-mediated cytokine secretion shown in our early study (10), whereas MCAM and PECAM are both involved in PNA-mediated effect shown in this study. The different profile of cytokine secretion, i.e. PNA induces the secretion of MCP-1 and IL-6 and galectin-3 induces secretion of IL-6, G-CSF, GM-CSF and ICAM-1 (10), is also in keeping with this conclusion.

PI3K-AKT-PREAS40 signalling was found to be the major signalling pathway involved in PNA-mediated MCP-1 and IL-6 secretion in this study. AKT is a serine/threonine kinase that is involved in multiple cell signalling pathways in divergent cell activities (42). AKT is often activated by upstream PI3K, one of the common signalling molecules pathogenically affected in cancer (43). AKT activation could lead to activation of multiple downstream signalling molecules such as GSK-3β, FOXO and mTORC in physiological and pathological conditions. One of the mTORC complex components is PRAS40. Phosphorylation of PRAS40 in response to AKT activation causes dissociation of PRAS40 from the mTORC1 complex, leading to mTORC1 activation (44). In this study, PNA-mediated secretion of MCP-1 and IL-6 and activation of AKT and PRAS40 were both abolished by the presence of PI3K inhibitors (Figure 6). This indicates that the PI3K-AKT-PRAS40 signalling axis is largely responsible for PNA-mediated actions in endothelial cells. PI3K activation by cell membrane CD80/CD86 activated AKT has been shown previously to induce IL-6 expression in dendritic cells (45). Activation of AKT and PRAS40 in intrahepatic cholangiocarcinoma by tumour-associated macrophages has been reported to lead to secretion of a number of cytokines including IL-6 and MCP-1 (46). These studies are all in keeping with our conclusion that PI3K-AKT-PREAS40 signaling is critical in PNA-mediated secretion of MCP-1 and IL-6.

Thus, in addition to its influence on promoting tumour cell spreading by interaction with tumour cell-associated MUC1 as reported in our earlier study (12), circulating PNA could also influence metastasis by enhancing the secretion of metastasis-promoting MCP-1 and IL-6 from the endothelium. The enhanced metastasis by PNA demonstrated in mice in our previous study (12) therefore likely represents a combination of the effect of PNA on tumour cells (12) as well as its effects on vascular endothelium.

It should be mentioned that in a previous case–control study, we reported a modest association (OR 1.37; 95% CI 1.01–1.85) between regular peanut consumption and colorectal cancer risk (47). However, a study based on cohorts from the Nurses’ Health Study and the Health Professionals Follow-up Study reported no significant impact of peanut consumption on cancer mortality (HR 0.94; 95%CI 0.88–1.02) (48). In another study, peanut consumption was reported to have no significant effect on prognosis in men with established prostate cancer (49). In our previous healthy volunteer study, the 5 µg/ml PNA blood concentration was only seen transiently 1 h or so after consumption of a large dose (250 g) of peanuts (3), so it may be that “normal” peanut consumption yielding lower PNA concentrations is harmless. Nevertheless, the possibility remains that circulating PNA, at least at the relatively high levels found shortly after a large “dose” of peanuts, could have a significant biological effect on tumour cells circulating at that time, with a potential for increased risk of metastasis.

## Abbreviations

ASF: asialo-fetuin
BSA: bovine serum albumin
Gal-3C: N-terminally truncated galectin-3
HMVE-Ls: human microvascular lung endothelial cells
HUVECs: human macrovascular umbilical vein endothelial cells
MCAM: melanoma cell adhesion molecule (CD146)
MCP-1: monocyte chemoattractant protein-1
NECDS: non-enzymatic cell dissociation solution
PNA: peanut agglutinin
